# Dietary supplementation of *Rosmarinus officinalis* L. leaves in sheep affects the abundance of rumen methanogens and other microbial populations

**DOI:** 10.1186/s40104-016-0086-8

**Published:** 2016-04-27

**Authors:** Gabriella Cobellis, Zhongtang Yu, Claudio Forte, Gabriele Acuti, Massimo Trabalza-Marinucci

**Affiliations:** Department of Veterinary Medicine, University of Perugia, via S. Costanzo 4, 06126 Perugia, Italy; Department of Animal Sciences, The Ohio State University, 2029 Fyffe Road, Columbus, OH 43210 USA

**Keywords:** Archaea, Essential oil, Plant extracts, Rosemary, Rumen microbiome

## Abstract

**Background:**

Rumen microbiome has a great influence on ruminant health and productivity. Different plant extracts have been tested for their ability to modulate the rumen microbiome to improve feed digestion and fermentation. Among the evaluated plant extracts, essential oils, tannins, and saponins appeared to have positive effects on rumen protein metabolism, volatile fatty acids production, and methane and ammonia production.

**Methods:**

The objective of this study was to evaluate the effect of rosemary (*Rosmarinus officinalis* L*.*) leaves and essential oils on rumen microbial populations. Four ruminally cannulated sheep were used in a 4×4 Latin square design fed (21 d/period): 1) a control diet composed of alfalfa hay and concentrate pellet (CTR), 2) CTR supplemented with 7 g/d/sheep of rosemary essential oil adsorbed on an inert support (EO), 3) CTR with 10 g/d/sheep of dried and ground rosemary leaves (RL), and 4) CTR with 10 g/d of dried and ground rosemary leaves pelleted into concentrate (RL pellet). Abundance of total bacteria, archaea, protozoa, and some select bacterial species or groups was quantified using qPCR, while the community of bacteria and archaea was profiled using denaturing gradient gel electrophoresis.

**Results:**

No difference in abundance was noted for total bacteria, protozoa, or *Ruminococcus flavefaciens* between the control and the treatments, but the rosemary leaves, either in loose form or in pellet, decreased the abundance of archaea and the genus *Prevotella* (*P* < 0.001). The rosemary leaves in loose form also decreased (*P* < 0.001) the abundance of *Ruminococcus albus* and *Clostridium aminophilum*, while the EO increased (*P* < 0.001) the abundance of *Fibrobacter succinogenes.* The community of bacteria and archaea was not affected by any of the supplements.

**Conclusions:**

Being able to affect the abundance of several groups of rumen microbes that are known to be involved in degradation of protein and fiber and production of methane and ammonia, rosemary leaves may be used to modulate rumen microbiome and its function.

## Background

The ruminant livestock sector contributes significantly to global emission of greenhouse gas (GHG) as methane and nitrous oxide, the latter of which is produced from the nitrogen (as urea and ammonia) excreted by ruminant animals [1, 2]. Both the methane and nitrogen outputs also represent a loss of dietary energy and nitrogen, which are otherwise redirected to animal production. Both methane emission and nitrogen excretion result from feed fermentation by rumen microbiome. Several compounds or substances have been tested as dietary supplements for their ability to modulate the composition and metabolic activities of rumen microbiome and to mitigate methane emission and nitrogen excretion from ruminant animals [1, 2]. Among them, plant extracts, such as essential oils, tannins, and saponins, seem to have positive effects on rumen protein metabolism, volatile fatty acids (VFA) production, methane and ammonia production [3–5]. However, the dilemma is that they often exert adverse effects on feed intake, digestion, and rumen fermentation when added at concentrations high enough to achieve substantial or desirable reduction in methane production, while they have little effect when added at permissive concentrations that do not reduce animal productivity [2]. New dietary intervention strategies are being sought after, including using combinations of different inhibitors that have additive inhibition to methane production by rumen microbiome [6, 7].

Rosemary (*Rosmarinus officinalis* L.) is an evergreen perennial shrub belonging to the *Lamiaceae* family and rosemary leaves are commonly used as a food seasoning. The secondary metabolites in rosemary are well known, with major compounds including monoterpenoids, such as *α*-pinene, *β*-pinene, camphene, *1*–*8* cineole, camphor, borneol, bornyl acetate and verbenone, and phenolic diterpenes, such as carnosol, carnosic acid, rosmanol, epirosmanol, isorosmanol, methyl carnosate, and rosmarinic acid [8]. Some of these compounds have antimicrobial and antioxidant activities. Several studies have evaluated rosemary essential oil as feed additive using in vitro rumen fermentation [9–13], but these studies focused on the effect on feed digestion, not methane production or ammonia production. In a previous study using sheep [8], the authors found that rosemary leaves pelleted into concentrate (RL pellet) contained less phenols, but more flavonoids, rosmarinic acid, and total antioxidant activity than the same rosemary leaves that were not pelleted (RL) and rosemary essential oil. Carnosic acid was detected in the RL pellet and the RL diets, but not rosemary essential oil. Rumen pH, VFA, and lactic acid concentrations of the sheep were not affected by RL, RL pellet, or rosemary essential oil, but CP and DM degradability and ammonia concentration (a tendency) were decreased by RL pellet [8]. The objective of this study was to evaluate the two forms of rosemary leaves (RL and RL pellet) and rosemary essential oil for their effect on select rumen bacterial populations and methanogens.

## Methods

### Animals, diets, experimental design, and sampling

The animals, diet, and feeding have been described previously [8]. Briefly, four Bergamasca x Appenninica ruminally cannulated sheep (6 years old, with a mean body weight of 60.5 ± 3.4 kg) were used in a replicated 4×4 Latin square design. The experimental procedures and animal care conditions were approved by the Bioethics Committee of the University of Perugia and authorized by the Italian Ministry of Health. The animals were kept in four individual pens, with each pen fed one of the four treatment diets: 1) 1.5 kg/d of alfalfa hay and 400 g/d of concentrate pellet (CTR), 2) CTR supplemented with 7 g/d of rosemary essential oil (EO) adsorbed onto inert material consisting of calcium carbonate and calcium and potassium aluminosilicate, 3) CTR with 10 g/d of dried and ground rosemary leaves (RL), and 4) CTR with 10 g/d of dried and ground rosemary leaves pelleted into concentrate (RL pellet). The diet composition was reported in Table 1. Feed was offered twice daily in equal meals (8:00 and 16:00 h), and each treatment lasted for 21 d. Different forms of rosemary supplementations (dry ground leaves, pelleted leaves, and essential oil extract) were evaluated because they differed in composition of secondary metabolites. Based on the rosemary EO content determined in a previous study [8], the amount of each form of rosemary supplementation fed to each sheep was calculated to give a dose of EO (0.05 g/kg of dry matter). To facilitate the introduction and mixing of the supplement in both the EO and RL diets, all feed ingredients were subjected to a rough grinding. Rumen content was sampled from each sheep before morning feeding from 3 different sites of the rumen after 21 days of adaptation on each diet and frozen immediately at −80 °C until analyses.

**Table 1:** Ingredients (% as fed basis) and chemical composition (g/100 g) of the concentrates used in the experimental diets (by Cobellis et al. [8])

Item	Diet			
Ingredients	CTR	RL pellet	RL	EO
Wheat bran	40.00	30.00	39.00	39.30
Wheat flour middlings	17.80	24.30	17.35	17.49
Corn grain	10.00	10.00	9.75	9.82
Sunflower meal	14.90	14.90	14.53	14.64
Soybean meal	5.00	6.00	4.87	4.91
Calcium carbonate	4.20	4.20	4.09	4.13
Dehydrated alfalfa meal	3.50	3.50	3.41	3.44
Beet protein concentrate	2.00	2.00	1.95	1.96
Sugar cane molasses	2.00	2.00	1.95	1.96
Vitamin-mineral supplement^1^	0.60	0.60	0.60	0.60
Rosemary leaves	-	2.50	2.50	-
Rosemary essential oil	-	-	-	1.75
Chemical composition				
*Analysed*				
Dry matter	92.88	92.78	92.84	92.96
Crude protein	18.40	18.44	18.09	18.08
Crude fat	3.08	3.21	3.12	3.05
Ash	9.84	9.98	9.79	11.24
NDF	29.65	29.52	29.79	29.13
ADF	10.65	11.17	10.99	10.47
Lignin sa	3.14	2.99	3.38	3.08
Ca	0.80	0.70	0.79	0.79
P	0.75	0.68	0.75	0.74
Na	0.28	0.27	0.27	0.28
*Calculated*				
Lys	0.70	0.71	0.68	0.69
Met	0.29	0.29	0.28	0.28
Met + Cys	0.58	0.58	0.56	0.57
Choline	0.14	0.14	0.14	0.14

^1^Supplied per kilogram of diet: Vitamin A, 18,000 I.U. (retinol); Vitamin D_3_, 2,100 I.U.; Vitamin E, 21 mg (α-tocopheryl acetate); Fe, 29 mg; Co, 0.75 mg; Mn, 39 mg; Zn, 150 mg; Se, 0.06 mg. CTR: control; RL pellet: CTR plus 10 g/d of dried and ground rosemary leaves pelleted into concentrate; RL: CTR plus 10 g/d of dried and ground rosemary leaves; EO: CTR plus 7 g/d of rosemary essential oil adsorbed on an inert support

### Metagenomic DNA extraction and quantitative real-time PCR analyses

The rumen samples were processed as described by Mosoni et al. [14]. Briefly, the frozen rumen content samples were thawed at 4 °C overnight. For each rumen sample, 5 g of solid phase and 5 g of liquid phases were combined with 10 mL of sterile distilled water and homogenized for 10 min using a Stomacher (PBI International, Milan, Italy). The homogenate was centrifuged at 6,500 × g at 4 °C for 30 min and the supernatant was discarded. Metagenomic DNA extraction was performed using 0.25 g of the pellet obtained after centrifugation according the method of Yu and Morrison [15]. DNA quality was evaluated using agarose gel (1 %) electrophoresis and DNA yield was quantified using NanoDrop 2000 (Thermo Scientific, Wilmington, USA). The DNA samples were stored at −20 °C until analysis.

All quantitative PCR (qPCR) analyses were performed on a Stratagene Mx3000p system (Stratagene Corporation, La Jolla, USA). Total bacterial population was quantified using a TaqMan assay, while the abundance of archaea, protozoa, and select bacterial species were quantified using SYBR Green-based specific qPCR assays. The primers and some of the PCR conditions used are shown in Table 2. One sample-derived qPCR standard was prepared for each target bacterial group using the respective specific PCR primer pair and a composite metagenomics DNA sample that was prepared by pooling an equal amount of all DNA samples as described previously [16]. After purification using a PCR Purification kit (Qiagen, Valencia, USA) and quantification using a Quant-iT dsDNA broad-range assay kit (Invitrogen, Carlsbad, USA), *rrs* gene copy number concentration of each qPCR standard was calculated from its length and the mass concentration. Tenfold serial dilutions of each purified standard were prepared in Tris-EDTA buffer prior to qPCR assays. In the SYBR-based qPCR assays, one 86 °C for 30 s step was added to each cycle and fluorescence signal was acquired at 86 °C to eliminate the effect from potential primer dimers [16]. Each qPCR assay was performed in triplicate for all samples and the respective qPCR standards using the same master mix and the same qPCR plate. The absolute abundance of each bacterial group was expressed as log_10_ of *rrs* gene copies/g of rumen content.

**Table 2:** Primers used to quantify ruminal microbes (qPCR) and to profile bacterial and archaeal communities (DGGE)

Organisms	Primers	Sequences (5′ → 3′)	Annealing temperature, °C	Amplicon length, bp	References
Real-time PCR					
Total bacteria	27f	AGA GTT TGA TCM TGG CTC AG	55	1535	[41]
	1525r	AAG GAG GTG WTC CAR CC			
Total bacteria	Eub358f	TCC TAC GGG AGG CAG CAG T	60	448	[42]
	Eub806r	GGA CTA CCA GGG TAT CTA ATC CTG TT			
	TaqMan probe	6-FAM-5′-CGT ATT ACC GCG GCT GCT GGC AC-3′-TAMRA	70		
Archaea	ARC787f	ATT AGA TAC CCS BGT AGT CC	60	272	[16]
	ARC1059r	GCC ATG CAC CWC CTC T			
Protozoa	316f	GCT TTC GWT GGT AGT GTA TT	54	223	[43]
	539r	CTT GCC CTC YAA TCG TWC T			
* Fibrobacter succinogenes*	Fs219f	GGT ATG GGA TGA GCT TGC	63	446	[44]
	Fs654r	GCC TGC CCC TGA ACT ATC			
* Ruminococcus flavefaciens*	Rf154f	TCT GGA AAC GGA TGG TA	55	295	[44]
	Rf425r	CCT TTA AGA CAG GAG TTT ACA A			
* Ruminococcus albus*	Ra1281f	CCC TAA AAG CAG TCT TAG TTC G	55	175	[44]
	Ra1439r	CCT CCT TGC GGT TAG AAC A			
* Prevotella spp.*	BAC303f	GAA GGT CCC CCA CAT TG	56	418	[45]
	BAC708r	CAA TCG GAG TTC TTC GTG			
* Clostridium aminophilum*	C.amin-57 F	ACG GAA ATT ACA GAA GGA AG	57	560	[46]
	C.amin-616R	GTT TCC AAA GCA ATT CCA C			
PCR-DGGE					
Total bacteria	GC-A357f	CCC TAC GGG GCG CAG CAG	61 → 56 °C	194	[17]
	519r	GWA TTA CCG CGG CKG CTG			
Archaea	GC-RC344f	ACG GGG YGC AGC AGG CGC GA	61 → 56 °C	191	[18]
	519r	GWA TTA CCG CGG CKG CTG			

FAM: 6-carboxyfluorescein; TAMRA: 6-carboxytetramethylrhodamine

### PCR-DGGE analysis

Denaturing gradient gel electrophoresis (DGGE) was used to evaluate the overall response of bacterial and archaeal communities to rosemary supplements as described previously [17, 18]. Briefly, the V3 hypervariable region of the 16S rRNA gene of bacteria and archaea was amplified using bacterium- and archaeon-specific primers, with a 40-bp GC clamp added to the 5′ end of the forward primer (Table 2). The PCR and DGGE conditions and the gel image analysis were essentially the same as described previously [19].

### Statistical analysis

All data were analysed as a 4 × 4 Latin square using the ANOVA procedure of SAS [20]. The statistical model included sheep, period, dietary treatment, and residual error. Fixed effects included period and dietary treatment. Sheep was the random effect. The abundances of rumen microbial populations (*rrs* gene copy number/g of rumen content) were first log-transformed prior to statistical analysis to improve normality. Overall differences between the means were evaluated using a Tukey test. Data were reported as least squares means ± standard error. Differences were considered to be significant when *P* ≤ 0.05. Based on the intensity and migration of the DGGE bands, a principal component analysis (PCA) was performed using the PC-ORD program as described by Patra and Yu [21] to analyze DGGE results.

## Results

### Abundance of rumen microbial populations

The results of the qPCR are shown in Table 3. Overall, the abundance of total bacteria, protozoa, and *Ruminococcus flavefaciens* was not affected by any of the rosemary supplements. The abundance of archaea and *Prevotella* spp. was, however, significantly decreased (*P* < 0.001) by the two diets containing rosemary leaves (RL or RL pellet). The RL diet also decreased (*P* < 0.001) the abundance of *Ruminococcus albus* and *Clostridium aminophilum*. The rosemary EO did not affect any of the quantified rumen microbial populations except for *Fibrobacter succinogenes*, which was increased (*P* < 0.001) compared to the control. The abundance of total archaea was decreased by both RL and RL pellet but not by the rosemary essential oil.

**Table 3:** Effects of different rosemary supplements on select rumen microbial groups (log_10_ *rrs* copies/g)

	Diet				SEM	*P*-value
	CTR	RL pellet	RL	EO		
Total Bacteria	11.03	11.01	10.89	11.01	0.06	0.1994
Archaea	8.86^a^	8.69^b^	8.64^b^	8.80^a^	0.05	<0.001
Protozoa	8.26^ab^	7.71^b^	7.97^b^	8.75^a^	0.15	<0.001
*Prevotella* spp.	9.92^a^	9.68^b^	9.72^b^	9.90^a^	0.12	<0.01
*Fibrobacter succinogenes*	6.86^b^	6.90^ab^	6.88^b^	7.00^a^	0.11	<0.001
*Ruminococcus albus*	7.62^a^	7.67^a^	7.27^b^	7.64^a^	0.16	<0.001
*Ruminococcus flavefaciens*	7.40^ab^	7.59^a^	7.59^a^	7.43^ab^	0.16	<0.05
*Clostridium aminophilum*	7.05^a^	7.16^a^	6.62^b^	7.11^a^	0.37	<0.001

^a,b^Means with different letters within a row differ significantly (*P* ≤ 0.05)

CTR control; RL pellet: CTR plus 10 g/d of dried and ground rosemary leaves pelleted into concentrate; RL: CTR plus 10 g/d of dried and ground rosemary leaves; EO: CTR plus 7 g/d of rosemary essential oil adsorbed on an inert support; *NS* not significantly

### Community profiles of bacteria and archaea

**Fig. 1:**
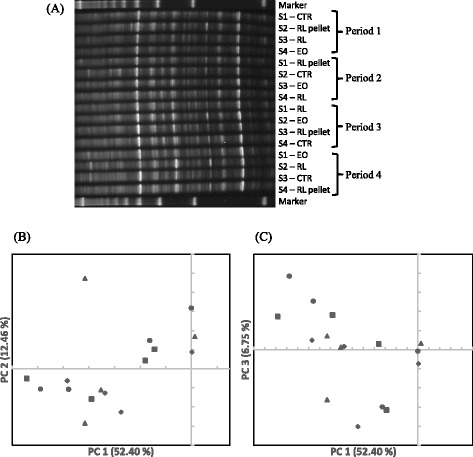
DGGE profiles (**a**) and PCA plots (**b** and **c**) of bacteria. S1, S2, S3, and S4: sheep 1, 2, 3, and 4, respectively. CTR (●): control; RL pellet (■): CTR plus 10 g/d of dried and ground rosemary leaves pelleted into the concentrate; RL (♦): CTR plus 10 g/d of dried and ground rosemary leaves; EO (▲): CTR plus 7 g/d of rosemary essential oil adsorbed on an inert support

The DGGE profile of bacteria showed a large number of bands and a complex pattern (Fig. 1a). Differences in intensity of some bands were noted, but little difference in banding patterns was visible between the control and the treatments, suggesting minimal effects of the rosemary supplements on the ruminal bacterial community of the sheep. The first three principal components (PC’s) together explained 71.61 % of the total variation (Fig. 1b and c). No clear separation of bacterial community profiles between the control and any of the treatments along the first principal component (PC1) axis that explained more than 50 % of the total variation. No separation of bacterial community profiles was seen along the second principal component (PC2) axis or the third principal component (PC3) axis, which explained 12.46 and 6.75 % of the total variation, respectively.

**Fig. 2:**
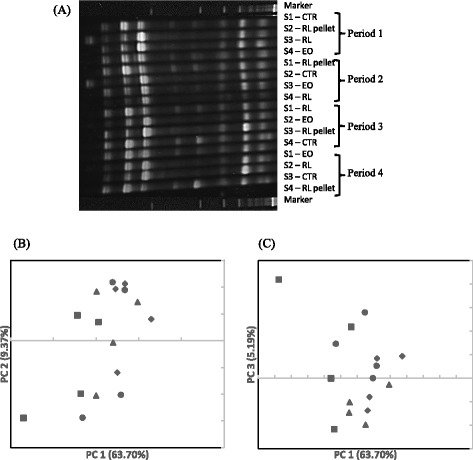
DGGE profiles (**a**) and PCA plots of archaea (**b** and **c**). S1, S2, S3, and S4: sheep 1, 2, 3, and 4, respectively. CTR (●): control; RL pellet (■): CTR plus 10 g/d of dried and ground rosemary leaves pelleted into the concentrate; RL (♦): CTR plus 10 g/d of dried and ground rosemary leaves; EO (▲): CTR plus 7 g/d of rosemary essential oil adsorbed on an inert support

The DGGE profiles of archaea showed a small number of bands, and no difference in number or intensity of bands was noticeable between the control and any of the treatments (Fig. 2a). The PC1, PC2, and PC3 together explained 78.26 % of the total variation, and no separation of the archaeal community profiles was evident among the four different diet groups (Fig. 2b and c).

## Discussion

In recent years, extracts from a variety of plants have been evaluated for their ability to modulate rumen microbiome, feed digestion, and rumen fermentation. Some plant compounds have been revealed to affect the abundance and/or the activity of rumen archaea, protozoa, and specific bacteria populations [5, 21–24]. Rosemary contains a number of antimicrobial monoterpenoids and phenolic diterpenes and rosemary supplements can potentially modify rumen functions. Their biological activity can be variable but several studies documented their specific activity on growth and energy metabolism of microbial cells [5].

A large number of in vitro and in vivo studies have been performed to test the ability of plant extracts to modulate rumen microbiome, to our knowledge; however, this is the first in vivo study to evaluate the effects of rosemary supplementations in different forms on rumen bacteria and archaea using cultivation-independent molecular biology techniques. In a previous study, we showed that rosemary leaves and essential oil decreased CP and DM digestibility and tended to lower rumen ammonia concentration in sheep [8]. In the present study, the abundance of *Prevotella* and protozoa (though only numerically) was lowered by rosemary leaves, ether in pellet (RL pellet) or in loose form (RL), and RL also decreased the population of *C. aminophilum* (Table 3)*.* Because members of *Prevotella* and protozoa are the mainly proteolytic microbes and *C. aminophilum* is one the three well documented hyper-ammonia-producing bacterial species [25], the decreased CP digestibility observed in the previous study [8] might be attributed to the decrease of these microbial groups. By the same token, the lower DM degradation could be related to the reduction of the abundance of *Prevotella* and *Ruminococcus albus* (Table 3).

The abundance of the analyzed microbial groups was affected by any of the rosemary leaves, but essential oil at the tested dose increased the abundance of *F. succinogenes* (Table 3). The differential effects between rosemary leaves and essential oil could be related to differences in the chemical composition of their antimicrobial compounds, with phenolic diterpenes, such as carnosic acid and rosmarinic acid, being rich in rosemary leaves, while rosemary essential oil is rich in monoterpenoids [8]. According to a number of studies [26–28], rumen microbes can adapt to essential oils, especially at low levels. One mechanism is reduction of active components of essential oils to inert alcohols by some microbes [29]. Indeed, Bernardes et al. [30] showed that the antimicrobial activity of extracts from rosemary leaves could be ascribed mainly to carnosic acid and carnosol, while rosmarinic acid has no antimicrobial activity against selected bacteria. However, a few studies showed that both rosmarinic and carnosic acids have antioxidant and antimicrobial activities [31, 32], and interestingly, rosemary extracts with similar rosmarinic acid content but different ratios of two phenolic diterpenes, carnosic acid and carnosol, differed in antibacterial activities. These observations suggest chemical interactions among different secondary metabolites and such interaction may affect the antimicrobial potency of rosemary extracts. In addition, some of the monoterpenoids in rosemary essential oil have a fairly broad range of antimicrobial activity [31, 33–35]. Some of these compounds are chemically instable and/or high volatile [36]. The lack of effect of the rosemary essential oil observed in the present study might result from loss of some instable or volatile antimicrobial compounds.

The rosemary leave supplementation, either in loose form or in pellet, decreased the abundance of archaea. However, the magnitude of the decrease in archaeal abundance is relatively small. Ohene-Adjei et al. [37] suggested that a reduction in the abundance of methanogen could be observed after prolonged inhibition of methane synthesis. Indeed, lack of decrease in archaeal abundance by anti-methane inhibitors has been observed in short-term in vitro incubation [21, 22, 38]. In addition, although not reaching statistical significance, the rosemary leaves also only lowered the abundance of rumen protozoa, potentially decreasing protozoa-associated methanogens and their contribution to methane production. Furthermore, the ability of rosemary leaves to decrease the abundance of *R. albus,* a hydrogen-producing bacterial species, points toward a potential to lower production of hydrogen, the electron donor of methanogenesis. Therefore, rosemary leaves, as suggested by some authors for other plant extracts [3, 37–39], may directly inhibit methanogenic archaea and inhibit some microbial metabolic processes contributing to methane production.

As revealed by DGGE analyses, no significant effect on the overall bacterial or archaeal communities was noted from any of the rosemary supplements at the tested doses (Figs. 1 and 2). The lack of apparent broad effect on bacterial or archaeal communities is consistent with the similar total bacterial abundance in the control and the rosemary treatments. DGGE can only detect some predominant members of microbial communities, and thus some of the affected members might have not been detected by the DGGE analysis. In addition, after a period of adaptation, some rumen microbes could acquire the capability of degrading and inactivating plant compounds [40], and variations among individual animals could also ‘hide’ dietary effects. However, many aspects about the relationship between dietary components and rumen microbiome are still poorly understood (such as similar fermentation characteristics of cows fed the same diet but with different rumen microbiome structure). For this reason, further efforts will be required to identify safe and effective compounds able to positively affect rumen microbial ecosystem and its fermentation, reducing the production of pollutants (methane and ammonia) by decreasing the abundance of the microbes that are involved in methane and ammonia production in the rumen.

## Conclusion

This study demonstrated that dietary supplementation with rosemary leaves can affect the abundance of several groups of rumen microbes that are known to be involved in degradation of protein and fiber and production of methane and ammonia. Given the potential effects on rumen fermentation, future studies are warranted to further evaluate rosemary supplementation in modulating rumen microbiome and modifying rumen function, especially methane production and nitrogen excretion.
